# Silk‐Gel Powered Adenoviral Vector Enables Robust Genome Editing of PD‐L1 to Augment Immunotherapy across Multiple Tumor Models

**DOI:** 10.1002/advs.202206399

**Published:** 2023-02-25

**Authors:** Ming Wu, Hao Li, Cao Zhang, Yingchao Wang, Cuilin Zhang, Yuting Zhang, Aoxue Zhong, Da Zhang, Xiaolong Liu

**Affiliations:** ^1^ The United Innovation of Mengchao Hepatobiliary Technology Key Laboratory of Fujian Province Mengchao Hepatobiliary Hospital of Fujian Medical University Fuzhou 350025 P. R. China; ^2^ The Liver Center of Fujian Province Fujian Medical University Fuzhou 350025 P. R. China; ^3^ Mengchao Med‐X Center Fuzhou University Fuzhou 350116 P. R. China; ^4^ Fujian Provincial Clinical Research Center for Hepatobiliary and Pancreatic Tumors Mengchao Hepatobiliary Hospital of Fujian Medical University Fuzhou 350025 P. R. China

**Keywords:** adenoviral vector, CRISPR/Cas9, immunotherapy, PD‐L1 gene editing, silk‐gel

## Abstract

Immune checkpoint blockade based on antibodies has shown great clinical success in patients, but the transitory working manner leads to restricted therapeutic benefits. Herein, a genetically engineered adenovirus is developed as the vector to deliver CRISPR/Cas9 (sgCas9‐AdV) to achieve permanent PD‐L1 gene editing with efficiency up to 78.7% exemplified in Hepa 1‐6 liver cancer cells. Furthermore, the sgCas9‐AdV is loaded into hydrogel made by silk fiber (SgCas9‐AdV/Gel) for in vivo application. The silk‐gel not only promotes local retention of sgCas9‐AdV in tumor tissue, but also masks them from host immune system, thus ensuring effectively gene transduction over 9 days. Bearing these advantages, the sgCas9‐AdV/Gel inhibits Hepa 1‐6 tumor growth with 100% response rate by single‐dose injection, through efficient PD‐L1 disruption to elicit a T cell‐mediated antitumor response. In addition, the sgCas9‐AdV/Gel is also successfully extended into other refractory tumors. In CT26 colon tumor characterized by poor response to anti‐PD‐L1, sgCas9‐AdV/Gel is demonstrated to competent and superior anti‐PD‐L1 antibody to suppress tumor progression. In highly aggressive orthotopic 4T1 mouse breast tumor, such a therapeutic paradigm significantly inhibits primary tumor growth and induces a durable immune response against tumor relapse/metastasis. Thus, this study provides an attractive and universal strategy for immunotherapy.

## Graphical abstract

Immune checkpoint blockade based on monoclonal antibodies often works in a transitory manner, which leads to restricted therapeutic benefits. This study presents a potent and durable CRISPR/Cas9 machinery through the corporation of adenoviral vector and silk‐gel for highly efficient PD‐L1 genome editing, to overcome the low response rate and drug resistance of current immune checkpoint blockades.

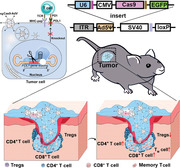

## 1 Introduction

Immune checkpoint blockade (ICB) therapy, as the most representative immunotherapy paradigm, has made breakthroughs in the field of tumor treatment. Especially, the monoclonal antibodies targeting programmed death‐1 (PD‐1) and programmed death‐ligand 1 (PD‐L1) are under the spotlight as the standard of care to display momentous clinical benefits in many cancers.^1^ However, the current response rates on solid tumors are limited (10–35%),^[^ ^1^, ^2^, ^3^ ^]^ and drug resistance is aggressively evolved in PD‐1/PD‐L1 blockades. Recent studies suggest that when PD‐L1 on the surface of tumor cells is blocked by PD‐L1 antibody, tumor cells will cleverly repopulate the intracellular PD‐L1 to cell surface to maintain a homeostasis state and finally mediate immune resistance/invasion.^[^ ^4^, ^5^ ^]^ Therefore, seeking potent alternatives to completely and permanently silence PD‐L1 expression of cancer cells would be a promising strategy to enhance ICB efficacy.

The clustered regularly interspaced short palindromic repeat‐associated endonuclease 9 (CRISPR/Cas9) has shown great promise as a powerful tool to edit genomes in a permanent and precise manner, which provides a reliable strategy to overcome the above issues that ICB blockades usually encounter.^[^ ^6^, ^7^, ^8^ ^]^ The CRISPR/Cas9 is commonly simplified by a single‐guide RNA (sgRNA) and a Cas9 endonuclease to form sgRNA/Cas9 complex to create site‐specific double‐strand breaks (DSBs) in the genome.^[^ ^9^, ^10^, ^11^, ^12^ ^]^ The breaks repaired by non‐homologous end joining thus lead to gene knockout.^[^ ^13^ ^]^ Due to sgRNA and Cas9 endonuclease are not naturally present in mammalians and are poor membrane permeable, safe and effective delivery of CRISPR/Cas9 components is necessary to advance their genome editing for in vivo applications. With respect to the in vivo disruption of PD‐L1 genome by CRISPR/Cas9, Deng et al. recently developed a biodegradable cationic polymer of poly(*β*‐amino esters) to deliver CRISPR/Cas9 plasmids target Cyclin‐dependent kinase 5 gene to reduce PD‐L1 expression in tumor cells, which demonstrated favorable therapeutic efficacy in mouse melanoma and breast cancer models.^[^ ^14^ ^]^ Other nonviral carriers such as supramolecular cationic gold nanorod,^[^ ^15^ ^]^ polyethylenimine derivatives,^[^ ^16^, ^17^, ^18^ ^]^ and mesoporous silica nanoparticle^[^ ^19^ ^]^ have also been exploited to deliver CRISPR/Cas9 components for PD‐L1 gene editing. Despite the simplicity and safety of nonviral carriers, they still suffer from the poor transduction and genome editing efficacy. In contrast, viral vectors are so far the most efficient vehicles to deliver various nucleic acid‐based therapeutics including CRISPR/Cas9 systems, and some of them have been approved for clinical uses. For example, CRISPR delivered by adeno‐associated viruses (AAVs) has recently been approved to eliminate the mutation on the CEP290 gene for the treatment of retinal degeneration (NCT03872479). Compared to AAVs with restricted packaging capacity (4.7 kb), adenovirus (AdV) has a large encapsulating size of 35 kb to easily fit the spCas9 discovered in *Streptococcus pyogenes* (the most widely used form of Cas9) with the sequence size above 4.2 kb.^[^ ^20^ ^]^ Meanwhile, AdVs scarcely integrate foreign genes into the host genome, thus avoiding the risk of oncogenicity or genotoxicity. These advantages, together with the minimal symptoms documented in clinical trials, make AdVs a suitable vector for delivery of spCas9 machinery, which has not been explored for in vivo PD‐L1 gene editing to amplify ICB efficacy so far.

Although these potential advantages, there are several issues related to viral vectors for gene delivery, such as nonspecific sequestration,^[^ ^21^, ^22^ ^]^ preexisting antiviral immunity, and the innate immune response,^[^ ^23^, ^24^ ^]^ which pose major concerns to develop viral vectors for in vivo application.^[^ ^25^ ^]^ To circumvent these obstacles, local administration is adopted in most of clinical trials using AdVs as gene therapy vectors.^[^ ^20^, ^26^ ^]^ In this case, viral release is deeply influenced by the local microenvironment, such as interstitial pressure, tissue gap, and the space between cells. For instance, numerous studies verified that the viral vector immediately appeared in other distant sites (liver and serum) following intratumoral administration, indicating severe diffusion issues related to the fenestrated or leaky tumor microvessels.^[^ ^27^, ^28^ ^]^ To reduce the systemic dissemination of viral vectors, considerable efforts have been devoted to employing biomaterials as versatile platforms to achieve effective and sustained release of viral vectors.^[^ ^29^, ^30^ ^]^ Among them, hydrogels have the advantages of adaptive rheological properties to closely contact with the surrounding tissues and mild fabrication conditions in aqueous solutions without disturbing viral vectors’ activity.^[^ ^31^ ^]^

**Figure 1:**
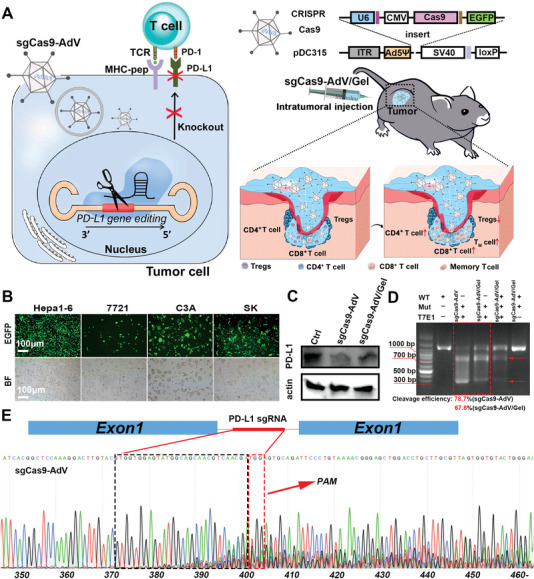
A) Schematic illustration of the construction of sgCas9‐AdV and sgCas9‐AdV/Gel for PD‐L1 editing to reprogram immunosuppressive tumor environment and enhance tumor immunotherapy. B) Fluorescence microscopy of EGFP expression in various cancer cells of Hepa 1‐6, SMMC‐7721, C3A, and SK cells, after infection with sgCas9‐AdV. C) Western Blot of PD‐L1 protein expression of Hepa 1‐6 cells after infection with sgCas9‐AdV or sgCas9‐AdV/Gel. D,E) The T7E1 assay (D) and Sanger sequencing (E) of PCR amplicon of the targeted PD‐L1 locus in Hepa 1‐6 cells after transduction with sgCas9‐AdV or sgCas9‐AdV/Gel.

As a biocompatible material, silk‐gel obtained from mulberry silk enables drug release in a sustained manner with limited immunogenicity and cytotoxicity, which has been employed by our group and others to enhance therapeutic effects through local injection.^[^ ^32^, ^33^ ^]^ Herein, we report a local CRISPR/Cas9 delivery system (termed sgCas9‐AdV/Gel) by loading the AdV vectors that encode spCas9 and PD‐L1 targeted sgRNA into an injectable silk‐gel. The silk‐gel protects sgCas9‐AdV from antibody neutralization by the host and enables their sustained release to improve gene editing efficiency and therapeutic efficacy (**Figure** **1**A). We characterize sgCas9‐AdV/Gel in terms of AdV release, transduction, and PD‐L1 gene editing efficiency, and further use this system to assess the therapeutic effect across multiple tumor models, which is demonstrated to be associated with the increased antitumor immune response but reduced antiviral immunity.

## 2 Results

### 2.1 Construction and Characterization of SgCas9‐AdV

In this study, human adenovirus serotype 5 (Ad5) vector, the best studied and most widely used serotype, was selected to equip CRISPR/Cas9. Specially, E1 gene was intentionally omitted to render Ad5 replication incompetent, thus ensuring its safety for in vivo applications. However, this modified virus can still replicate in specific human cell lines like 293A in which E1 is naturally present. Thus, recombinant AdV was produced in 293A cells through co‐transfection of the shuttle plasmid of pDC315 inserted with sgRNA/Cas9 and backbone plasmid pBHGlox (delta) E1, 3Cre by using Lipofectamine 3000 (Figure 1A). The adenoviral supernatants were purified by CsCl gradient centrifugation to obtain recombinant CRISPR/Cas AdV particles (termed sgCas9‐AdV). The titer of sgCas9‐AdV vectors was determined by a quantitative PCR (qPCR) assay (Figure S1, Supporting Information). From image of transmission electron microscopy (TEM), sgCas9‐AdV showed a typical icosahedral capsid of known structure with a diameter of 70–90 nm (Figure S2, Supporting Information). To explore adenoviral vector‐induced transgene expression in vitro, sgCas9‐AdV vectors containing enhanced green fluorescent protein (EGFP) cassette were incubated with different types of cancer cells. As displayed by fluorescence microscopy in Figure 1B, most cancer cells treated with the AdV at a multiplicity of infection (MOI) of 300 were EGFP‐positive, indicating robust infection capacity of adenoviral vectors to a variety of tumor cells including murine hepatoma cell line of Hepa 1‐6, human hepatoma cell lines of SMMC‐7721 and C3A, as well as human neuroblastoma cell line of SK. These results suggest the high potential of Ad5 as a vector to deliver sgCas9 in multiple cancer cells for gene editing.

Next, the Hepa 1‐6 cell line was selected as a model to investigate gene editing effect by checking PD‐L1 expression through Western Blot. As expected, the expression of PD‐L1 was obviously down‐regulated in Hepa 1‐6 cells after infection with sgCas9‐AdV for 48 h (Figure 1C and Figure S3, Supporting Information). To further verify that the PD‐L1 disruption is associated with genome editing, indel frequency was detected by T7 endonuclease 1 (T7E1) digestion assays. For this purpose, we designed a pair of primers about 300 bp upstream and 700 bp downstream of the targeted PD‐L1 gene for indel analysis by performing nucleic acid electrophoresis in a 1% agarose gel. Under the action of the T7E1 enzyme that recognizes and cuts incompletely matched DNA fragments generated from the indels (deletion or insertion) during non‐homologous end joining after CRISPR/Cas9 based genome editing,^[^ ^15^, ^34^ ^]^ the sgCas9‐AdV‐treated cells (WT−Mut + T7E1+ group) showed distinguishable cut bands at 300 and 700 bp from the uncut bands at 1000 bp around the genomic locus of PD‐L1, with the indel frequency determined to be 78.7% from quantifying gray intensities of these bands (Figure 1D). In addition, Sanger sequencing profile of PD‐L1 loci revealed two or more peaks around the protospacer adjacent motif (PAM) by Ad5 mediated CRISPR‐Cas9 transduction (Figure 1E). Collectively, the results suggest that the adenoviral vectors are constructed successfully, and are very competent to mediate the transduction of CRISPR/Cas9 for effective PD‐L1 gene editing in vitro.

### 2.2 sgCas9‐AdV Loaded into Silk‐Gel for Gene Transduction In Vivo

**Figure 2:**
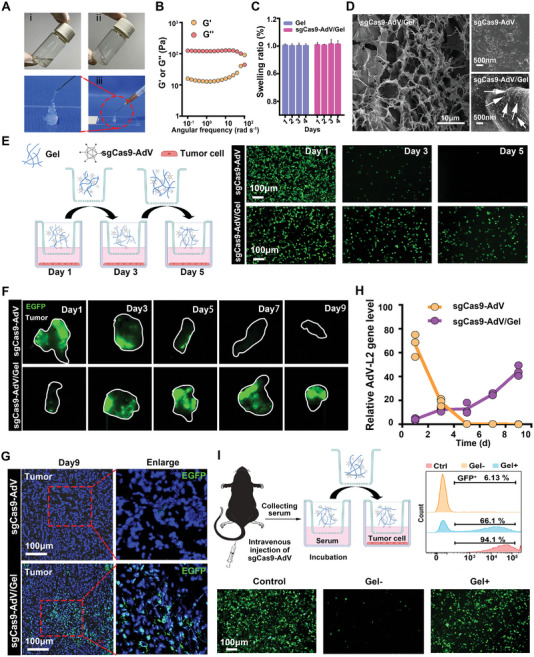
Preparation and characterization of sgCas9‐AdV/Gel. A) Representative photographs of the silk fiber before (i) and after (ii) ultrasound treatment for gel formation, as well as the solid‐like hydrogel depot through syringe injection (iii). B) Frequency‐dependent rheological properties of silk‐gel. C) Swelling properties of silk‐gel before and after loading with sgCas9‐AdV. D) Scanning electron microscopy of sgCas9‐AdV and gCas9‐AdV/Gel. E) A transwell system to investigate the release of the virus from silk‐gel to infect cancer cells in vitro. F) EGFP fluorescence signal in tumor tissues after intratumoral injection of sgCas9‐AdV and sgCas9‐AdV/Gel at different time points. White circles indicated tumor area. G) The representative fluorescence images of the frozen tumor sections after intratumoral injection of sgCas9‐AdV and gCas9‐AdV/Gel at the day 9. H) RT‐qPCR analysis of the intratumoral sgCas9‐AdV genomic DNA levels at the defined time points. I) Schematic diagram of neutralizing antibody experiments, which employed fluorescence microscope and flow cytometry to analyze the infection efficiency of sgCas9‐AdV and gCas9‐AdV/Gel after inactivated by the serum containing neutralizing antibody. The cells infected by sgCas9‐AdV without serum interference served as the positive control.

Even through local injection, conventional viral vectors can still disseminate through systemic circulation.^[^ ^27^ ^]^ Another downside is the risk of neutralizing antibodies to AdVs, which restrict their clinical use mostly in immune‐privileged tissues via local injection.^[^ ^35^ ^]^ To address these issues, we employed silk‐gel to improve the transduction efficiency of sgCas9‐AdV through avoiding virion dissemination and inactivation/elimination by preexisting antivirus immunity after local injection. Silk‐gel was prepared according to our previous methods,^[^ ^32^ ^]^ by placing the vial of silk fiber aqueous solution (3 wt%) in an ultrasound bath for 3 min to induce the change of molecular conformation from random coils to *β*‐sheets, and finally form hydrogels (**Figure** **2**A). Meanwhile, the obtained hydrogel was demonstrated to have certain flow ability to pass through a 26‐gauge needle and could form the pre‐designed geometric shapes (Figure 2A and Figure S4, Supporting Information), which is favor for local administration. In addition, the much higher of storage modulus (G′) than loss modulus (G″) also verified gel formation (Figure 2B). After loading with virions, the swelling properties of silk‐gel (sgCas9‐AdV/Gel) did not change, which showed a stable pattern after storing for more than 4 days (Figure 2C). To visually evaluate the spatial distribution of viruses in the hydrogel, sgCas9‐AdV/Gel was photographed by scanning electron microscopy (SEM). As depicted in Figure 2D, sgCas9‐AdV/Gel had a distinct porous and network microstructure uniformly filled with AdV virion, whose size was in line with that observed by TEM. In contrast, pure silk‐gel exhibited a smooth surface without virion embellishment (Figure S5, Supporting Information). In addition, confocal laser scanning microscope (CLSM) also verified the homogeneous distribution of AdV (labeled with Dylight 550) in silk‐gel (Figure S6, Supporting Information). Western Blot and T7E1 assay confirmed that sgCas9‐AdV/Gel maintained PD‐L1 gene editing capacity but with a slight less efficiency than free sgCas9‐AdV (Figure 1C,D), because of their slow release from silk‐gel to infect Hepa 1‐6 cells for gene editing. As a qualitative method, Sanger sequencing also intuitively verified the PD‐L1 gene editing effect of sgCas9‐AdV/Gel (Figure S7, Supporting Information). To prove the sustained release behavior, we have investigated the release profile of sgCas9‐AdV (labeled by DyLight 550) from silk fiber‐made hydrogel by spectrofluorometric assay. As shown in Figure S8, Supporting Information, sgCas9‐AdV/Gel presented a slow and sustained release manner, in which the complete virus release need a much long period up to 5 days.

Next, we employed a trans‐well system to investigate the release of the virus from silk‐gel to infect cancer cells in vitro. The intended workflow is to place sgCas9‐AdV/Gel into the upper chamber, while the lower chamber is seeded with Hepa 1‐6 cancer cells (Figure 2E). Observation by fluorescence microscopy verified the sustained release of virions from silk‐gel to continuously infect cancer cells in different plates for more than 5 days, in comparison with a strong EGFP fluorescence signal observed only on the 1st day in free sgCas9‐AdV group due to the fast diffusion of virion. This result suggests that silk‐gel can serve as a biocompatible reservoir to prolong AdV release without affecting its activity (Figure 2E). Encouraged by this result, we further compared the infectivity of the sgCas9‐AdV/Gel and free sgCas9‐AdV in tumor tissue after local injection. Ex vivo imaging of dissected tumors at different post‐injection times confirmed that sgCas9‐AdV/Gel could achieve a stable transgene expression of EGFP lasting up to 9 days, while free sgCas9‐AdV only induced transient transgene expression probably ascribing to the non‐specific dissemination and host elimination (Figure 2F). The sgCas9‐AdV transduction reinforced by silk‐gel was also supported by CLSM of tumor sections (Figure 2G). Accordingly, we found that the AdV‐associated DNA (indicated by L2 DNA^[36]^) gradually increased in the tumor tissues sampling apart from the injection site from day 1 to day 9 after intratumoral injection of sgCas9‐AdV/Gel, but showed a dramatically decreased curve as for the free sgCas9‐AdV group (Figure 2H). These results prove that the silk hydrogel can delay the release of the virus in vivo and increase the infection time of the tumor tissue. To further demonstrate this behavior, we also investigated the bio‐distribution of virus (labeled by ICG‐NHS) after intra‐tumoral injection (Figure S9, Supporting Information). At different time‐points of post‐injection, the tumors and major visceral organs were isolated for ex vivo fluorescence imaging. After 1 day of injection, free sgCas9‐AdV showed a homogeneous distribution in the whole tumor area, indicating its rapid diffusion from injection site. In addition to tumor site, a substantial fluorescence signal of sgCas9‐AdV also appeared in liver, which is consistent with previous reports that viral vectors easily leak from tumor and enter into liver through the fenestrated or leaky tumor microvessels.^[^ ^27^, ^28^ ^]^ However, sgCas9‐AdV/Gel exhibited a very different distribution profile, with only a part of tumor region around injection site presenting strong fluorescence signal due to the slow release of sgCas9‐AdV from silk‐gel. After 3 days of injection, the signal in tumor treated by free sgCas9‐AdV sharply decreased, which was also found to much lower than that in sgCas9‐AdV/Gel group. With the observation time further prolonged to 5 days, no virus fluorescence signal could be found in the tumor tissue and major organs of free sgCas9‐AdV treated mouse, suggesting the clearance of these free virus from body. In sharp contrast, the fluorescence signal in tumor site of sgCas9‐AdV/Gel group still displayed a strong pattern even after 5 days of injection. These results verified that our proposed silk‐gel could achieve sustained virus release in vivo to minimize their leakage into other tissues.

Another mechanism likely attributing to the superior transgene expression of sgCas9‐AdV/Gel in vivo is that the silk‐gel is capable to shield the virus from neutralizing antibodies. In order to confirm this hypothesis, we collected serum from the mice immunized with AdV as a source of neutralizing antibodies, and performed AdV vector transduction in a culture medium supplemented with this serum, according to the workflow illustrated in Figure 2I. From fluorescence microscope images, we found that when co‐incubated with serum in advance, the infectivity of sgCas9‐AdV (Gel− group) significantly decreased owing to the serum‐medicated inactivation of AdV vectors,^[^ ^21^, ^37^ ^]^ but silk‐gel effectively hid the sgCas9‐AdV from neutralizing antibodies in serum, as testified by the much higher expression of EGFP than naked free sgCas9‐AdV (Figure 2I). To further quantify the difference between these two groups, we measured the percentage of EGFP positive cells by flow cytometry, while the cells infected by sgCas9‐AdV without serum interference served as the positive control. As shown in Figure 2I, the EGFP positive rate of sgCas9‐AdV/Gel (Gel+) group reached 66.1%, while naked free gCas9‐AdV (Gel−) group was only 6.3%, which was in accordance with fluorescence microscope. In addition, we found that the encapsulation of AdV vector into silk‐gel could also decrease the generation of neutralizing antibodies in vivo (Figure S10, Supporting Information).

### 2.3 sgCas9‐AdV/Gel Mediated PD‐L1 Gene Editing to Elicit Anti‐Tumor Immunity

**Figure 3:**
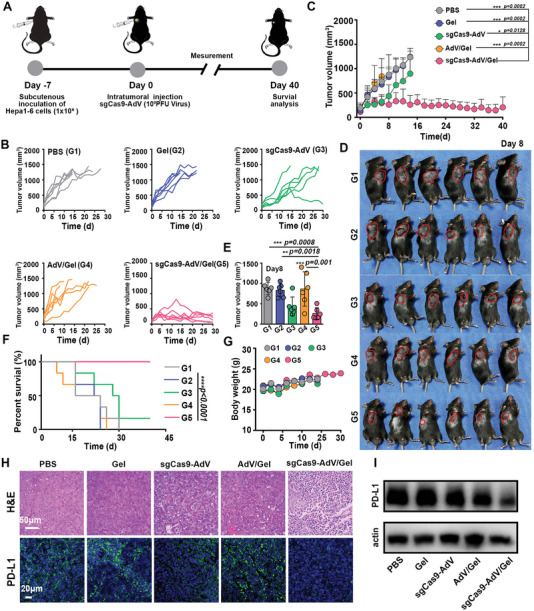
Therapeutic efficacy of sgCas9‐AdV/Gel on Hepa 1‐6 tumors. A) Scheme of the therapeutic procedure. B) Individual and C) average tumor growth kinetics in different groups. Growth curves were stopped when the tumor volume of the corresponding group exceeded 1500 mm^3^. D,E) Photograph of Hepa 1‐6 tumor‐bearing mice (D) and tumor volume (E) on the 8th day after receiving different treatments as indicated. The tumor area was labeled with a red circle. F) Kaplan–Meier survival curves and G) body weight change of Hepa 1‐6 tumor‐bearing mice after receiving different treatments as indicated. H) HE and PD‐L1 immunofluorescence (IF) staining of tumor slices for different treatment groups. I) Western Blot analysis of PD‐L1 expression in the tumors with indicated treatments. Data are presented as mean ± SD (*n* = 6).

Encouraged by the superior localized transduction and PD‐L1 genome editing effect of sgCas9‐AdV/Gel, we next explored its therapeutic potential in Hepa 1‐6 tumor xenografts (**Figure** **3**A). To this end, Hepa 1‐6 cells were injected into the armpit of immune‐competent C57BL/6 mice to establish a subcutaneous tumor model. When tumors grew to about 100 mm^3^ after 7 days of cancer cell inoculation, the mice experienced different treatments via intratumoral injection, such as PBS (G1), silk‐gel (Gel, G2), sgCas9‐AdV (G3), silk‐gel loaded with mock AdV that also bear CRISPR/Cas9 expression cassette but targeting an irrelevant sequence (AdV/Gel, G4), and sgCas9‐AdV/Gel (G5). As shown in Figure 3B,C, the tumor volume from the groups of PBS, Gel, and AdV/Gel showed a similar trend of rapid growth, with more than 80% of the mice exceeding the ethical limit within 20 days after treatment, which proved that the hydrogel and mock AdV vector alone could not inhibit tumor growth. In comparison, sgCas9‐AdV showed slight antitumor effects to delay tumor growth, but was unable to induce tumor regression. This result indicates that the naked free CRISPR/Cas9 AdVs are incompetent to inhibit tumor progression due to being rapidly cleared by the body after intratumoral injection. Most significantly, the sgCas9‐AdV/Gel displayed the strongest therapeutic effect compared with other groups. Photograph (Figure 3D) and quantified tumor volume at day 8 (Figure 3E) also revealed that sgCas9‐AdV/Gel substantially attenuated Hepa 1‐6 tumor growth by 70% (in tumor volume), significantly more efficient than the free sgCas9‐AdV group. At the end of the observation period (day 40), all the mice receiving only one‐shot injection of sgCas9‐AdV/Gel maintained alive with low tumor burden, whereas mice in other groups finally succumbed to tumors with limited survival benefits (Figure 3F). Meanwhile, none of the mice showed obvious body weight loss and damage to major organs during sgCas9‐AdV/Gel treatments (Figure 3G and Figure S11, Supporting Information), indicating the limited side effects of the sgCas9‐AdV/Gel. In contrast, some serum biochemical indicators reflecting liver functions, such as AST and ALT became abnormal when the mice were administrated with naked free sgCas9‐AdV (Figure S11, Supporting Information), probably ascribing to the diffusion of AdV from tumor to liver which cause hepatotoxicity (Figure S9, Supporting Information).^[^ ^38^ ^]^ Taken together, these results document the high potential of silk‐gel, as a biocompatible platform to improve therapeutic efficiency of AdV‐based CRISPR/Cas9 system targeting PD‐L1.

We further performed a pathological analysis of tumor tissues of mice to verify the therapeutic effect after different treatments as indicated above. The results of hematoxylin and eosin (HE) and Ki67 staining of tumor sections confirmed that there were severe damages in the mice treated with sgCas9‐AdV/Gel (Figure 3H and Figure S12, Supporting Information). Accordingly, the protein expression of PD‐L1 was dramatically down‐regulated by sgCas9‐AdV/Gel‐mediated genome editing as detected by immunofluorescence and Western Blot analyses (Figure 3H,I and Figure S13, Supporting Information), suggesting the great promise of silk‐gel and AdV to corporately deliver CRISPR/Cas9 system to induce PD‐L1 disruption for therapeutic applications.

**Figure 4:**
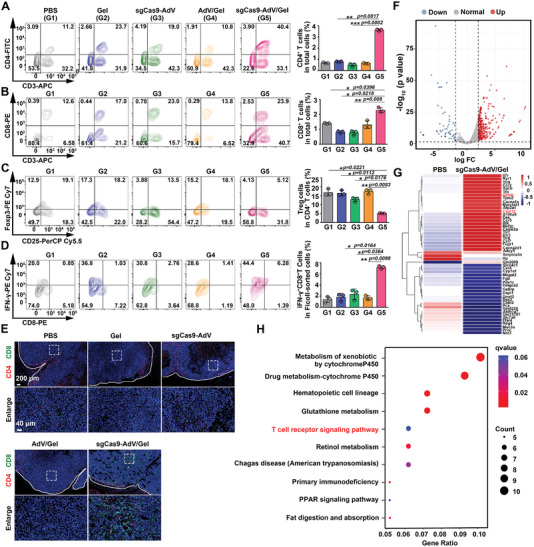
Evaluation of intratumoral immune response. A–D) The percentage of tumor‐infiltration of CD3^+^CD4^+^ T (A), CD3^+^CD8^+^ T (B), Treg (C), and IFN*γ* ^+^CD8^+^ T (D) cells after receiving different treatments were analyzed by flow cytometry on the 4th day. Data are presented as mean ± SD (*n* = 3). E) Immunofluorescence staining of tumor infiltrated CD4^+^ T cells (red) and CD8^+^ T cells (green) on the 4th day after receiving different treatments. F–H) scRNA‐seq analysis of the tumor tissues from mice after treatment. F) Volcano plot for DEGs with PBS and sgCas9‐AdV/Gel treatments. G) Heatmap of the top 25 most DEGs with PBS and sgCas9‐AdV/Gel treatments. H) KEGG enrichment analysis of the DEGs between PBS and sgCas9‐AdV/Gel treatments.

Having proved the impressive antitumor effect, we subsequently explored the corresponding immune response in this tumor model. First, the immune cell subsets in the tumor tissue were analyzed from Hepa 1‐6 tumor‐bearing mice on the day 5 post aforementioned treatments. Obviously, sgCas9‐AdV/Gel injection elevated the levels of CD3^+^CD4^+^ (helper) and CD3^+^CD8^+^ (cytotoxic) T cells in tumor tissues (**Figure** **4**A,B and Figure S14, Supporting Information), while reducing the infiltration of Tregs (Figure 4C and Figure S15, Supporting Information). Meanwhile, the percentage of IFN‐*γ* ^+^CD8^+^ T cells was the highest in the sgCas9‐AdV/Gel‐treated group (Figure 4D and Figure S15, Supporting Information), thus potentiating the adaptive immune response for tumor control. The content of TNF‐*α* and INF‐*γ* in tumor tissues also showed that the sgCas9‐AdV/Gel treatment elicited a significant immune response (Figure S16, Supporting Information). Observation of the tumor section by immunofluorescence staining further confirmed the most enrichment of tumor‐infiltrating CD4^+^ T cells and CD8^+^ T cells after sgCas9‐AdV/Gel treatment (Figure 4E).

In order to further explore the function pathways related to treatment response, the differentially expressed genes (DEGs) in the tumor tissues of PBS and sgCas9‐AdV/Gel groups were analyzed by transcriptome RNA sequencing (scRNA‐seq). Volcano plots of DEGs in Figure 4F presented that 1050 transcripts were substantially differentially expressed after sgCas9‐AdV/Gel treatment; of these DEGs, 117 were downregulated and 933 were upregulated. DEGs coupled with Kyoto Encyclopedia of Genes and Genomes (KEGG) enrichment analysis revealed that some biological processes were associated with immune response, such as upregulation of granzyme B (GZMB) and chemokine CXCL12, as well as enrichment in the T cell receptor signaling pathway (Figure 4G,H).

**Figure 5:**
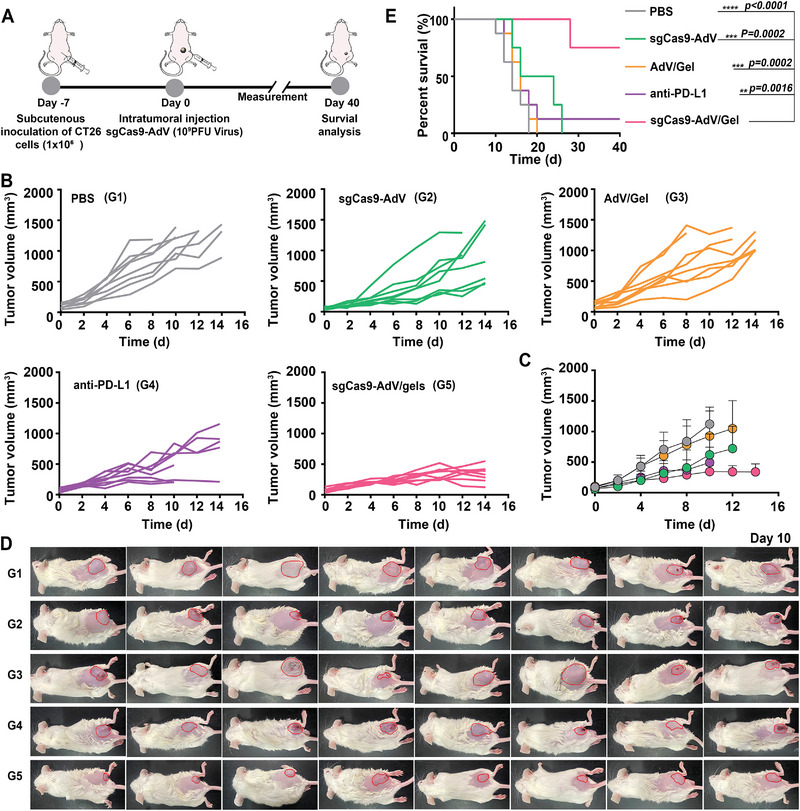
Evaluation of the therapeutic effect on CT26 tumors. A) Scheme of the therapeutic procedure for CT26 colon tumor. B) Individual and C) average tumor growth kinetics in different groups. Growth curves were stopped when the tumor volume of the corresponding group exceeded 1500 mm^3^. Data are presented as mean ± SD (*n* = 8). D) Photograph of CT26 tumor‐bearing mice on the 10th day after receiving different treatments as indicated. The tumor area was labeled with a red circle. E) Kaplan–Meier survival curves of mice with indicated treatments.

Encouraged by the above results, we further assessed the therapeutic efficacy of sgCas9‐AdV/Gel in CT26 colon murine tumor model, which is characterized by poor response to anti‐PD‐L1 based ICB.^[^ ^39^ ^]^ The treatment schedule was similar to those for Hepa 1‐6 liver cancer therapy, that is, only one intra‐tumoral injection of sgCas9‐AdV/Gel (**Figure** **5**A). Meanwhile, we added PD‐L1 antibody as control which was intraperitoneally injected into tumor‐bearing mice every 2 days for four times. As expected, AdV/Gel without CRSPR/Cas9 system did not lead to any tumor growth suppression or survival benefit. The sgCas9‐AdV alone could moderately delay tumor growth and enhanced overall survival to some degree. Notably, the sgCas9‐AdV/Gel substantially inhibited tumor progression and prolonged survival time, with six out of eight mice maintained alive during the whole observation period up to 40 days, whereas anti‐PD‐L1 exhibited much less ability to suppress tumor growth or extent overall survival of mice (Figure 5A–E). The superior antitumor effect of sgCas9‐AdV/Gel than anti‐PD‐L1 can be attributed to the permanent silence of PD‐L1 expression to overcome the immune resistance/invasion of cancer cells through repopulating the intracellular PD‐L1 to cell surface.

**Figure 6:**
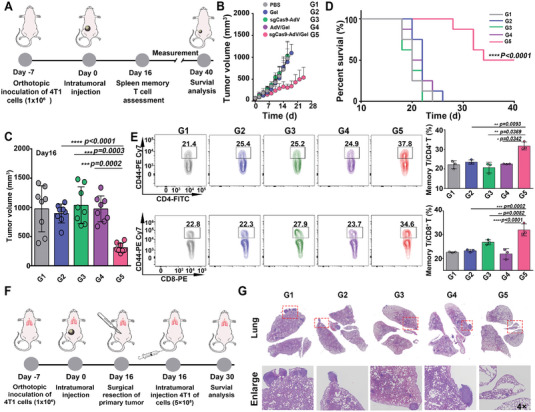
Evaluation of the therapeutic effect on 4T1 tumors. A) Scheme of the therapeutic procedure for orthotopic 4T1 breast cancer model and the detection of memory T cells in the spleen. B) Average tumor growth kinetics and C) tumor volume detected at the 16th day after receiving different treatments as indicated. Data are presented as mean ± SD (*n* = 8). D) Kaplan–Meier survival curves. Data is presented as mean ± SD (*n* = 8). E) The percentage of memory T cells of CD4^+^CD44^high^, and CD8^+^CD44^high^ T in the spleen after receiving different treatments that were analyzed by flow cytometry on the 16th day. Data are presented as mean ± SD (*n* = 3). F,G) Schematic illustration of sgCas9‐AdV/Gel treatment for lung metastasis inhibition of 4T1 tumor in Balb/c mice (F) and histological H&E staining of lung metastasis slices 30 days after different treatments (G). The enlarged area was indicated as a red dashed box, while tumor nodules were labeled with a blue circle.

In another orthotopic 4T1 mouse breast tumor model with highly aggressive and poorly immunogenic characteristics,^[^ ^40^, ^41^ ^]^ our sgCas9‐AdV/Gel also showed a better antitumor effect than other treatments (Figure 6A–D and Figure S17, Supporting Information). To further confirm whether sgCas9‐AdV/Gel could induce a durable immune response, the spleens were collected from the mice after treatments and then analyzed by flow cytometry (**Figure** **6**E). The mice that received sgCas9‐AdV/Gel exhibited a significant increase in the percentages of memory T cells (CD8^+^CD44^high^ and CD4^+^CD44^high^) in comparison with other groups. Consequently, much less tumor metastasis was observed in their lungs after intravenous re‐challenge of cancer cells (Figure 6F,G), suggesting the generation of long‐lasting anti‐tumor immune response. Together, these results prove that our sgCas9‐AdV/Gel elicit robust anti‐tumor immunity against multiple established mouse tumors and activate the body's long‐term anti‐tumor immunity against tumor relapse/metastasis.

## 3 Discussion

ICB targeting PD‐1/PD‐L1 pathway has shown remarkable prospects in cancer treatment, but also faces the bottlenecks of low response rate and adaptive drug resistance.^[^ ^42^ ^]^ Existing antibody‐based blockades only temporarily target to the cell surface exposed PD‐1/PD‐L1, while the newly expressed and intracellular cytoplasm storied PD‐1/PD‐L1 proteins would further repopulate to cell surface to mediate immune evasion.^[^ ^5^ ^]^ The gene editing technology based on the CRISPR/Cas9 system to specifically cleave the target gene sequence is expected to achieve permanent elimination of PD‐L1 in cancer cells, but its efficiency is closely related to the delivery vector. Viral vectors have become the first choice for delivering CRISPR/Cas9 system due to their wide infectivity and high efficiency in exogenous gene transduction.^[^ ^43^ ^]^ In this work, we selected replication‐deficient Ad5 as the vector to deliver CRISPR/Cas9 system. First, the AdV vector was characterized in vitro, confirming that the CRISPR/Cas9 system could be successfully transduced into various cancer cells (Hepa 1‐6, SMMC‐7721, C3A, SK) by virtue of the high invasiveness of Ad5, which is superior to that reported by non‐viral delivery vehicles for PD‐L1 editing, such as cationic gold nanorods and polyethylenimine derivatives.^[^ ^15^, ^16^ ^]^

However, the implementation of Ad5 for in vivo delivery is a severe challenge, due to its poor retention in the target tissue and pre‐existing of host immune responses.^[^ ^38^ ^]^ To address these issues, we selected the natural product of silk‐gel as a biocompatible material to improve the in vivo performance of sgCas9‐AdV based on our previous knowledge regarding to the application of silk‐gel.^[^ ^32^ ^]^ We demonstrated the advantages of loading sgCas9‐AdV into silk‐gel from multiple aspects, such as overcoming the challenge of non‐specific dissemination, masking the adenoviral particles from neutralizing antibodies, and avoiding dramatic antiviral activation of host immune system. Compared with the aggressively pursued strategy of genetically engineering or surface cloaked AdV to evade innate immunity, that needs complicated procedures and may affect virus activity,^[^ ^38^, ^44^ ^]^ our silk‐gel based strategy presented here has great advantages of lower cost and easy to prepare without damaging encapsulated therapeutic agents. Although various types of hydrogel have been used to encapsulate viral vectors to improve their in vivo performance, the silk fibroin in this work has unique advantages including tunable mechanical properties, superior biocompatibility, a simple fabrication process, and controllable processing parameters.^[^ ^45^ ^]^

Furthermore, permanent silence of PD‐L1 by a single‐dose local injection of our sgCas9‐AdV/Gel shows excellent efficacy to control tumor growth across multiple murine cancer models, through boosting persistent antitumor immune responses. Although the local administration route which was commonly adopted by virus‐based therapy seems not impressive comparing to systemic delivery in terms of the access to disseminated tumors, our sgCas9‐AdV/Gel treatment generating systemic immune responses in the host to fight against tumor metastasis remedies this imperfection in some degree. In this regard, more clinically relevant advanced tumor models, such as primary tumors with synchronous distant metastases should be applied to further validate the therapeutic potential in future studies. Moreover, even though local injection and silk‐gel encapsulation, it is difficult to completely confine the adenoviral vectors only in tumor site, but not in the liver (which is the major organ to sequester AdV); thus, despite our preliminary results confirmed that the silk‐gel encapsulation could reduce the hepatotoxicity of AdV, the influence of potential nonspecific gene editing in liver is worth of further study. Therefore, coupled with the tumor‐specific promoter to control CRISPR/Cas9 expression is also an effective approach to avoid nonspecific editing, which could be adopted in future studies to further improve the security of our proposed paradigm for PD‐L1 genome editing. In addition, further efforts in terms of optimizing the silk‐gel structure to balance gene editing and degradation of the scaffold in the body will help to advance the clinical translation.

## 4 Conclusion

In conclusion, this study presented a potent and durable CRISPR/Cas9 machinery through the corporation of adenoviral vector and silk‐gel for highly efficient PD‐L1 genome editing, which provides the possibility for overcoming the low response rate and drug resistance of current immune checkpoint blockades.

## 5 Experimental Section

Experimental details are all clarified in Supporting Information.

## Conflict of Interest

The authors declare no conflict of interest.

## Supporting information

Supporting Information

## Data Availability

Research data are not shared.
