# Supplementary material for: Silk‐Gel Powered Adenoviral Vector Enables Robust Genome Editing of PD‐L1 to Augment Immunotherapy across Multiple Tumor Models
Source: Adv Sci (Weinh). 2023 Feb 25;10(12):2206399. doi: 10.1002/advs.202206399 (PMC10131848; doi:10.1002/advs.202206399)
Supplement: Supplementary file 1 — Supporting Information [file ADVS-10-2206399-s001.pdf]

ADVANCED SCIENCE
Open Access

Supporting Information

for *Adv. Sci.*, DOI 10.1002/advs.202206399

Silk-Gel Powered Adenoviral Vector Enables Robust Genome Editing of PD-L1 to Augment Immunotherapy across Multiple Tumor Models

Ming Wu, Hao Li, Cao Zhang, Yingchao Wang, Cuilin Zhang, Yuting Zhang, Aoxue Zhong, Da Zhang and Xiaolong Liu**

Supporting Information

Materials

Plasmid pX458 was purchased from Addgene (USA). Plasmid pDC315 was obtained from Chinese Academy of Sciences. Plasmid pBHGlox (delta) E1, 3Cre was purchased from GeneBiogist Technology (Shanghai, China). The guide RNA and primers were synthesized by BIOSUNE Biological Technology (Shanghai, China). Bbs1, FastDigest XbaI (Cat. No. ER1011), FastDigest BamHI (Cat. No. FD0684), and T4 DNA ligase (FD0054) were purchased from Thermo Fisher. T7E1 was purchased from New England Biolabs, Inc. (Cat. No. M0302S). Lipofectamine 3000 Transfection Reagent was purchased from Thermo Fisher (Cat. no. L3000015). The anti-PD-L1 antibody (Cat. no. ab213480) and anti-β-actin antibody (Cat. no. ab179467) for Western Blot were purchased from Abcam. The phenylmethylsulfonyl fluoride (PMSF) and protease inhibitor cocktail were purchased from MedChemExpress (USA). Anti-PD-L1 antibodies for IHC and in vivo antitumor evaluation were purchased from Cell Signaling Technology (Cat. no. 64988T) and Biolegend (Cat. no. 64988T), respectively. The Albumin Bovine V was purchased from Solarbio (Cat. no. A8020) The antibodies for flow cytometry analyses (anti-CD3-APC, anti-CD4-FITC, anti-CD8-PE, anti-CD4-FITC, anti-CD25-PerCP-Cy5.5, anti-Foxp3-PE-Cy7, and anti-IFN-γ-PE-Cy7) were purchased from eBioscience (USA). IFN-γ and TNF-α ELISA kits were purchased from BOSTER Biological Technology (Wuhan, China). Mouse adenovirus antibody

(IgG) ELISA kit was purchased from CUSABIO (Cat. no. CSB-E13901m). Fetal bovine serum (FBS) was purchased from ExCell Bio (Shanghai, China).

sgCas9-AdV shuttle plasmid construction

The U6-sgRNA-CMV-Cas9-EGFP cassette (6301 bp in total) was amplified from pSpCas9(BB)-2A-EGFP (PX458) plasmid. Following purification with a DNA extraction kit (Omega Biotek, USA), U6-sgRNA-CMV-Cas9-EGFP cassette was inserted into the adenovirus shuttle plasmid (pDC315) by using XbaI/BamHI restriction enzymes (37°C, 1-2 h) and T4 DNA ligase (16°C, 4-6 h). Subsequently, a sgRNA sequence targeting mouse PD-L1 gene was cloned into above established plasmid with BbsI restriction enzyme to generate sgCas9-AdV shuttle plasmid (pDC315-U6-sgPD-L1-CMV-Cas9-EGFP). Positive clones were identified by DNA sequencing.

The primer sequence:

F: -5′GCTCTAGAGAGGGCCTATTTCCCATGATTCCT3′-

R: -5′GCGTCGACCTTGTACAGCTCGTCCATGCC3′-

The sgRNA sequence for targeting PD-L1 gene:

F: -5′GTATGGCAGCAACGTCACGA 3′-

R: -5′TCGTGACGTTGCTGCCATAC 3′-

Production of adenoviral vectors

HEK293A cells were seeded in 10 cm dishes at a density of 2×10^6 cells per dish one day before transfection, the adenoviral shuttle plasmid of pDC315-u6-sg-CMV-Cas9 and backbone plasmid of pBHGlox (delta) E1, 3Cre were

co-transfected into the cells with Lipofectamine 3000 transfection reagent. When cytopathic effect was observed after 3-5 days of transfection, the cells were ruptured by repeatedly liquid nitrogen freeze-thawing, and then centrifuged at 2000×g for 15 min at 4°C. Afterwards, the supernatant containing primary virus was used to infect fresh HEK293A cells to amplify the virus for three rounds. At last, the supernatant containing abundant viral particles were purified by CsCl gradient centrifugation to obtain sgCas9-AdV and stored at −80°C. To determine the titer of virus, adenoviral genome was extracted using TIANamp Virus DNA/RNA Kit (Tiangen Biotech， Cat. no. DP315) and subsequently analyzed by RT-qPCR. The morphology of sgCas9-AdV was observed by transmission electron microscope (FEI Tecani G20 TWIN) at the electron microscope laboratory at Wuhan Institute of Virology, CAS. The sample of sgCas9-AdV was prepared on copper grids by negative staining with uranyl acetate before being imaged by TEM.

Cell culture

HEK293A cells (human embryonic kidney cell line 293A), SMMC-7721 and C3A cells (human hepatocellular carcinoma cell line), SK cells (human neuroblastoma cell line), Hepa1-6 cells (mouse hepatocellular carcinoma cell line), CT26 cells (mouse colon carcinoma cell line), and 4T1 cells (mouse breast carcinoma cell line) were grown in complete RPMI-1640 or Dulbecco's Modified Eagle medium, respectively, supplemented with 10% fetal bovine serum, 2mM L-glutamine, 1% (v/v) penicillin/streptomycin. All cells were maintained at 37 °C in a humidified incubator

with 5% (v/v) CO_2. Cell lines were confirmed to be the absence of mycoplasma using HANBIO SaveIt™ detection kit.

In vitro adenoviral vector transduction

EGFP fluorescence was used to evaluate the transduction efficiency of sgCas9-AdV. In a typical experiment, the cells (Hepa1-6, SMMC-7721, C3A, SK) were seeded in 6-well plates at a density of 3×10^5 cells per well one day before infection with the sgCa9-AdV (10^7 pfu per well). After 48 h of transduction, the cells were examined for EGFP fluorescence by fluorescence microscopy (Zeiss Axio Vert.A1, Germany).

Animals

Six-week-old C57BL/6 and Balb/c mice were ordered from Shanghai China Wushi, Inc. (Shanghai, China). Animal experimental protocols were approved by the Animal Ethics Committee of Mengchao Hepatobiliary Hospital of Fujian Medical University and carried out according to the institutional guidelines.

T7 endonuclease I（T7E1）assay and Sanger sequencing

Hepa1-6 cells were seeded in 6-well plates at a density of 3×10^5 cells per well one day before infection with the sgCa9-AdV. The cells were collected by centrifugation at 300×g for 5min after infection for 48h. The cellular DNA was extracted using the TIANamp Genomic DNA Kit (Tiangen Biotech， Cat. no. DP304-3). Afterwards, a pair of PCR primers targeting PD-L1 gene about 300 bp upstream and 700 bp downstream were designed for indel analysis. The annealed PCR products were digested with T7 Endonuclease I and run on a 1% agarose gel,

finally analyzed with an imaging system (ChemiDoc™MP, Bio-Rad). The indel efficiency was calculated according to the following formula: (ii+iii)/(i+ii+iii) × 100%, where i is the intact band around 1000 bp, ii and iii are cleavage bands around 300 bp and 700 bp, respectively. The gray value of these bands was quantified using Image J.[1] In addition, PCR products of adenovirus-infected cells were subjected to Sanger sequencing.

Primer sequence:

F: -5´ CTGAACATTCCCAGGGAGGTG 3´-

R: -5´ CAAGCGAATCACGCTGAAAGTC3´-

Western Blot analysis

Cell lysates were prepared in RIPA lysis buffer (Beyotime Biotechnology, China) containing PMSF and a protease inhibitor cocktail (MedChemExpress, China), and the protein content of the generated cell lysates was determined using the BCA protein assay (TransGen Biotech, China). Aliquots containing 20 μg of total protein were loaded on SDS-polyacrylamide gels and transferred to nitrocellulose membranes. After membranes were blocked with 5% BSA for 1 h, they were probed with anti-PD-L1 antibody at a dilution of 1:1000 and anti-β-actin antibody at a 1:5000 dilution. After washing, the membranes were incubated with the HRP-conjugated secondary antibodies (1:5000) for 1h at room temperature. Antibody binding was detected using an imaging system (ChemiDoc™MP, Bio-Rad).

Synthesis of sgCas9-AdV/Gel

Silk-gel was obtained according to our previously published works.[2] For loading

with sgCa9-AdV, 1 mL of silk fibroin solution (3 wt%) was sonicated by an ultrasonic probe (Scientz-IID, Ningbo Scientz Biotechnology, China) with a duty cycle of 50% for 180 s, and then immediately mixed with 4 mL of adenovirus particles through a syringe, followed by rested at room temperature for gel formation at the adenovirus particles' concentrations of 2×10^{10} pfu/mL (sgCa9-AdV). The morphology was monitored by scanning electron microscopy imaging (Nova NanoSEM 230) at an accelerating voltage of 6 kV.

Evaluation of rheological and swelling properties

For evaluating the rheological property, the hydrogel was placed in a dynamic shear rheometer (M301) to perform rheology experiments. The storage modulus (G′) and loss modulus (G″) of hydrogels were determined at appropriate strain and stress. For evaluation of swelling property, the silk-gel and sgCas9-AdV/gel were respectively placed in PBS, and the weight of each group was measured once every day. The swelling ratio was calculated by dividing the measured weight by the initial weight of gel.

Sustained release in vitro

The silk-gel containing sgCas9-AdV was placed in a cell strainer with a pore size of 8 μm. Then, the strainer was embedded in a 24-well plate cultured with 1×10^{5} of Hepa 1-6 cells. Every 24 hours, the bottom of the strainer was washed 2-3 times with sterile PBS before transferred to another 24-well plate cultured with uninfected 1×10^{5} of Hepa1-6 cells in advance. At last, the cells were photographed by a fluorescent microscope to determine viral release.

Sustained release in vivo

To establish subcutaneous Hepa1-6 tumors, 3×10^6 murine Hepa1-6 cells in 100 μL of sterile PBS were injected subcutaneously into the armpit of C57BL/6 mice. When tumors reached an average size of 100 mm^3, the mice were divided into two groups and injected intratumorally with sgCas9-AdV and sgCas9-AdV/Gel, respectively. The amount of virus injected into the two groups of mice was 1×10^9 pfu. The mice were sacrificed at different time points to dissect the tumor tissue to observe the expression of EGFP in tumor tissue. At the same time, the expression level of the adenovirus L2 gene in the tumor tissue was detected by qPCR to determine the efficiency of virus release and infection.

Neutralizing antibody test

Anti-adenovirus antiserum was prepared as follows. C57BL/6 mice were injected with 1×10^9 pfu of adenovirus through tail vein on day 0 and day 20. Seven days later, the animals were sacrificed and the blood was collected through heart puncture. After 15min at room temperature, the blood was centrifuged at 4 °C for serum separation. The serum was then diluted with culture medium and incubated with sgCas9-AdV or sgCas9-AdV/Gel for 1 h at 37 °C, followed by being transferred into another 24-well plate pre-seeded with Hepa 1-6 cells to monitor the adenovirus infectivity by fluorescence microscope and flow cytometer.

For evaluating neutral antibody levels in vivo, C57BL/6 mice bearing Hepa 1-6 tumor were intratumorally injected with sgCas9-AdV or sgCas9-AdV/Gel. At the 1, 3, 5, 7, and 9 days post-injection, the neutralizing antibody levels in blood were detected

by ELISA Kit of mouse adenovirus antibody (IgG) (CSB-E13901m, CUSABIO).

Antitumor evaluation on subcutaneous Hepa1-6 and CT26 tumor model

For the establishment of subcutaneous Hepa1-6 tumors, 2×10^6 of Hepa1-6 cells in 100 μL of sterile PBS was injected s.c. into the armpit of C57BL/6 mice. When the tumors reached an average size of about 100 mm^3, the mice were randomized into different groups of PBS, silk-gel (Gel), sgCas9-AdV (1×10^9 pfu), AdV/Gel (1×10^9 pfu), and sgCas9-AdV/gel (1×10^9 pfu) through intratumoral injection with a volume of 50 μL. The tumors were measured by using a vernier caliper and the volume (V) was calculated to be $V=a\times b^2/2$, where a and b are the longest and shortest diameter of the tumor, respectively. Meanwhile, the mouse weights were also recorded during the treatment course. The mice were sacrificed when the tumor volume exceeded 1500 cm^3, or when the health condition of mice was impaired seriously.

According to similar methods, CT26 subcutaneous tumor model established on Balb/c mice was also employed to evaluate the antitumor effect of the aforementioned formulations. In addition, PD-L1 antibody as control was administrated by intraperitoneal injection (0.8 mg/kg) every two days for a total of four times.

Immune cell isolation and flow cytometry analysis

To analyze tumor infiltrated immune cells, the tumor tissues were harvested post-treatment and cut into small pieces, digested with collagenase IV (1 mg/mL), hyaluronidase (0.2 mg/mL), and Dnase I (0.02 mg/mL) for 2 h at 37°C. The single-cell suspension was obtained after filtering through a 40 μm filter. A part of the single-cell suspension was stained with anti-CD3-APC, anti-CD4-FITC and

anti-CD8-PE for the analysis of T cell infiltration. The remaining single-cell suspension was subsequently collected by centrifugation at 800×g for 5 min, and further purified by the Ficoll-Paque density gradient centrifugation. Next, the harvested cells were permeabilized and examined by flow cytometer after staining with anti-CD4-FITC, anti-CD25-PerCP-Cy5.5 and anti-Foxp3-PE-Cy7 for Tregs analysis, and with anti-CD8-PE and anti- IFN-γ-PE-Cy7 for IFN-γ^{+}CD8^{+} T cell analysis. All antibodies were purchased from eBioscience.

Histological evaluation

Tumors were collected from mice on day 4 after the indicated treatments and stained with hematoxylin and eosin (H&E), Ki67 and PD-L1. Tumor sections were also subjected to immunofluorescence staining for CD4 and CD8.

Cytokine analysis for tumor lysate

Tumors were isolated from mice on day 4 after treatment. 20-30 mg of tissue pieces were lysed in RIPA lysis buffer (Beyotime Biotechnology, China) containing protease inhibitor cocktail (MedChemExpress, USA), then homogenized with magnetic beads at 60 Hz for 180 s and centrifuged at 16,000×g for 10 min at 4°C. IFN-γ and TNF-α in the supernatants were detected by ELISA (Boster Biological Technology, China) according to the manufacturer's instructions.

Antitumor evaluation on orthotopic 4T1 tumor model

1×10^{6} 4T1 cells were inoculated on the Balb/c mice mammary gland. When the volume of tumor reached about 100 mm^{3}, the mice were randomly divided into 5 groups for the following treatments. PBS, silk-gel (Gel), sgCas9-AdV (1×10^{9} pfu),

AdV/Gel (1×10^9 pfu), and sgCas9-AdV/gel (1×10^9 pfu) were administrated through intratumoral injection with a volume of 50 μL. The tumor size was measured as mentioned above.

For isolation and analysis of memory T cells, three mice in each group were sacrificed on day 16 after different treatments, and the spleens were dissected. Splenocytes were passed through a 40 μm filter and isolated by Ficoll-Paque density gradient centrifugation before being stained with the corresponding antibodies and examined by flow cytometry. The antibodies used for flow cytometry are listed below: Anti-CD3-APC, anti-CD4-FITC, anti-CD8-PE, and anti-CD44-PE-Cy7.

Immune memory effect for inhibiting tumor lung metastasis

Orthotopic 4T1 tumor-bearing mice received above mentioned treatments. On day 16, residual tumors were surgically removed from the mice, and the wounds were sutured. At the same time, 5×10^5 4T1 cells were injected into mice through the tail vein. After approximately 2 weeks, lung tissue was isolated from mice with different treatments for H&E staining.

RNA sequencing and bioinformatic analysis

The tumor tissues were extracted and quickly frozen into liquid N_2. Subsequently, total RNA was extracted using Triol reagent kit according to the manufacturer's protocol. After extraction of total RNA, mRNA was further enriched and fragmented, and cDNA was obtained by reverse transcription. Then, transcriptome libraries were further constructed according to the manufacturer's instructions, and sequenced by the Illumina novaseq6000 platform (paired end, 150 bp).

All qualified raw reads were aligned to the mouse genome (mm10, https://www.gencodegenes.org) using STAR,[3] then expression levels of genes were further quantified using FPKM (transcripts per million) value and differential expression analysis between different groups was conducted by Cufflinks v2.2.1,[4] and KEGG pathway enrichment analysis was conducted with clusterProfiler package using differentially expressed genes (At the same time to meet the following two conditions: first. In the two groups of samples, the FPKM is greater than 0.5, or when the FPKM of one sample is 0, the FPKM of the other group should be greater than 1. Secondly, the Fold change is greater than 1.5 or less than 0.6667).

Statistical Analysis

Data were presented as mean ± standard deviation (SD). The data were analyzed by one-way analysis of variance (ANOVA) or t-test as indicated. The log-Rank test was used to compare survival differences. Prism 6 software (GraphPad) was used to perform all statistical analyses. Results were considered statistically significant when $*P<0.05$, $**P<0.01$, $***P<0.001$, $****P<0.0001$.

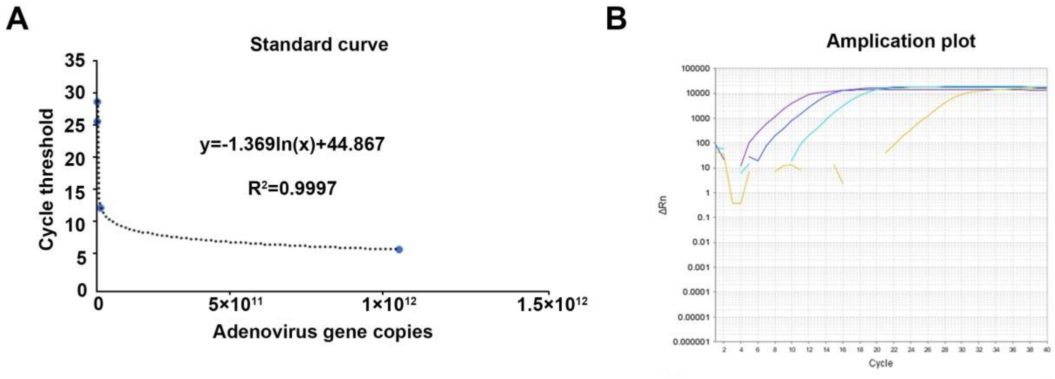

Figure S1. (A) Standard curve for determination of adenovirus titer by qPCR; (B) Amplification curves of standard adenovirus L2 gene with different copies by qPCR.

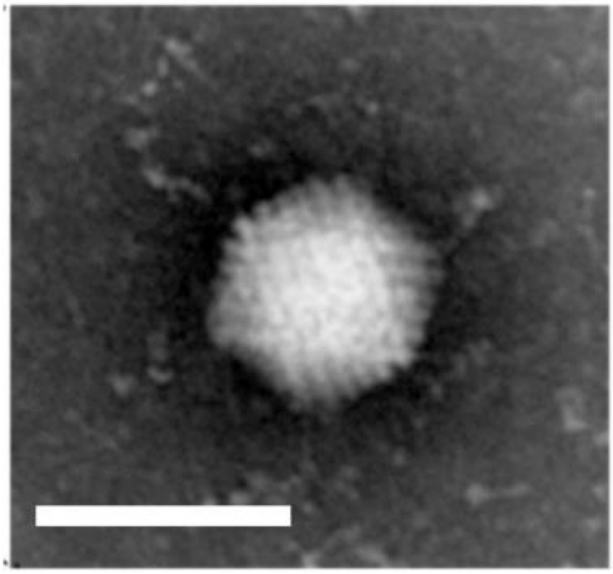

Figure S2. TEM image of sgCas9-AdV. Scale bar: 100 nm.

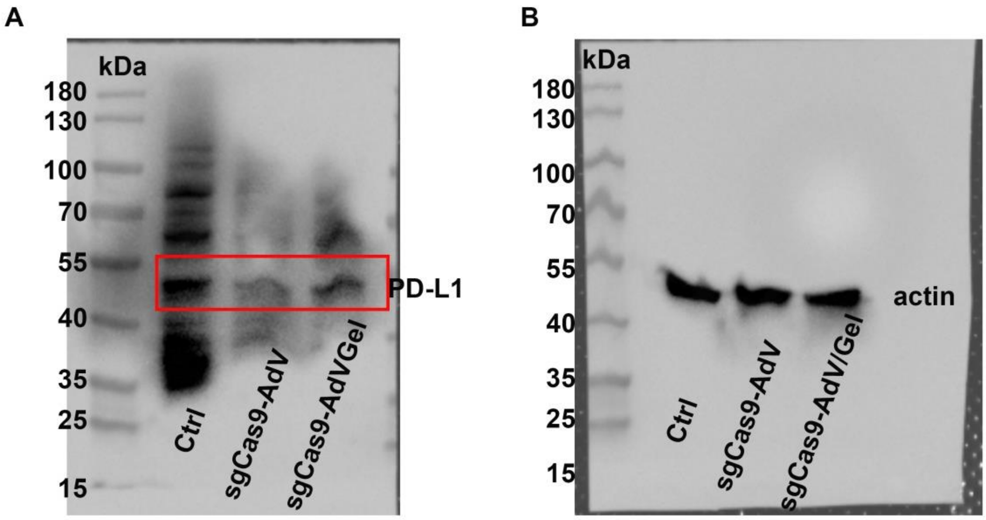

Figure S3. Full gel scan images of Figure 1C.

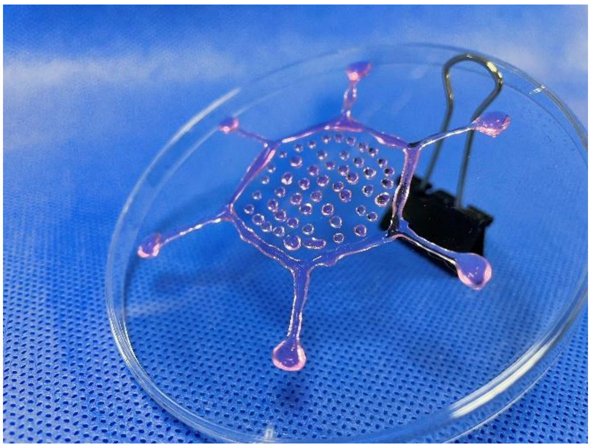

Figure S4. Representative photographs of the hydrogel and the formation of pre-designed geometric shapes after syringe injection. A robust, solid-like hydrogel depot is formed that does not flow because of gravity.

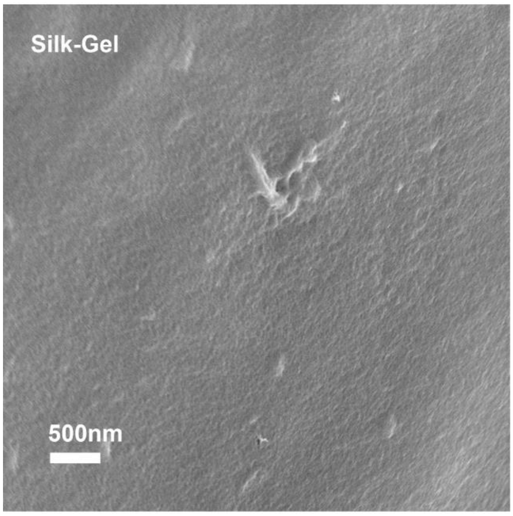

Figure S5 SEM image of silk-gel without AdV.

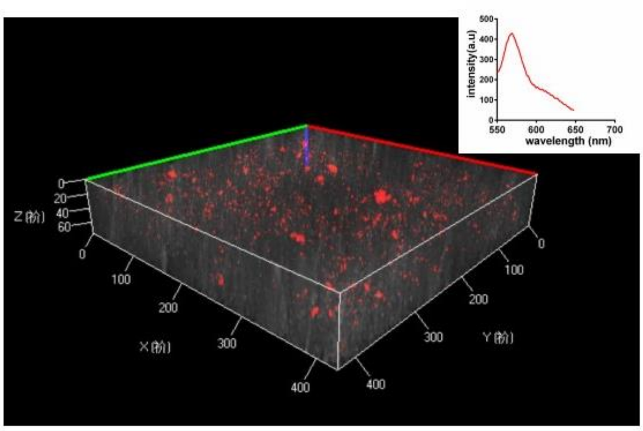

Figure S6. 3D CLSM image of CRISPR/Cas9 adenovirus labeled with DyLight 550 inside silk-gel. The inset image in the upper right corner shows the fluorescence spectrum of DyLight 550.

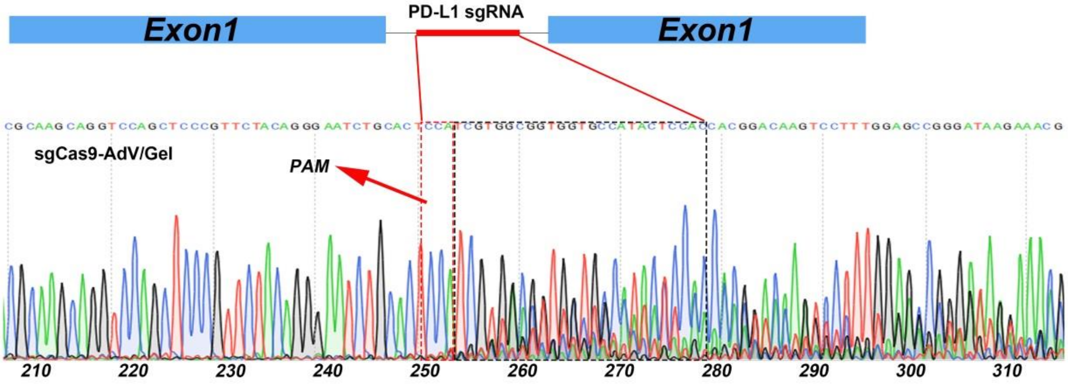

Figure S7. Sanger sequencing (E) of PCR amplicon of the targeted PD-L1 locus in Hepa1-6 cells after transduction with sgCas9-AdV/Gel.

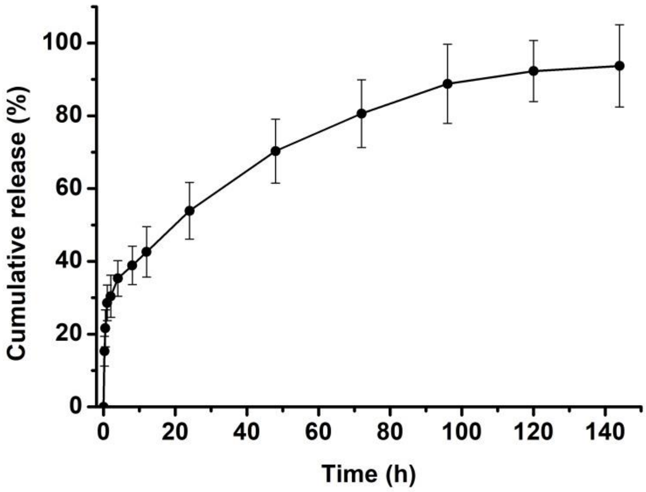

Figure S8. Cumulative sgCas9-AdV release from silk-gel.

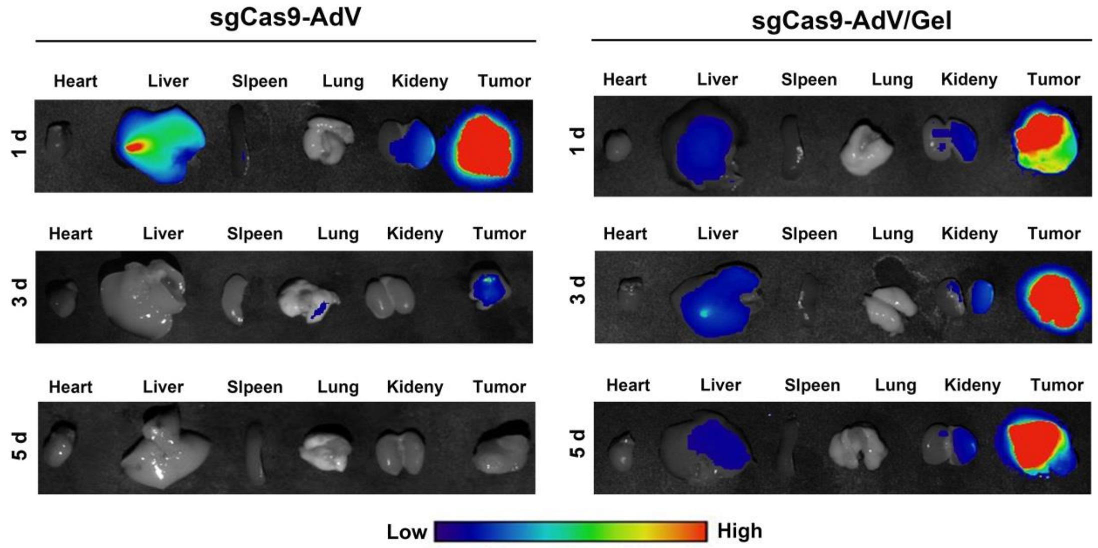

Figure S9. Ex vivo images of visceral organs and tumors isolated from the mice after intratumoral injection of sgCas9-AdV and sgCas9-AdV/Gel at different time points, which were acquired by a NIR II fluorescence imaging system (UNITED WELL).

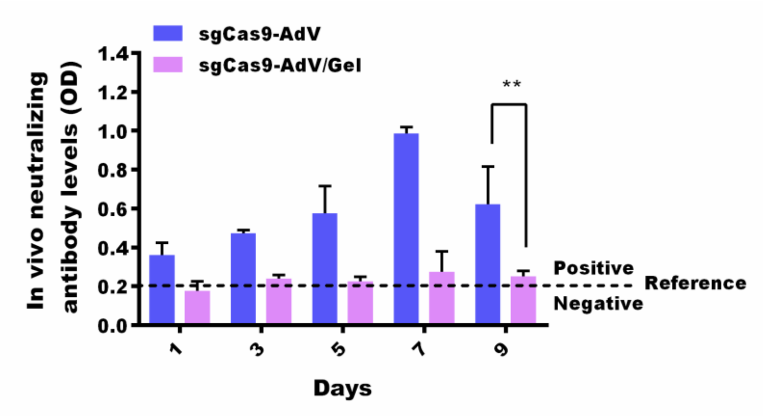

Figure S10. ELISA detection of the neutralizing antibody level in serum after intratumoral injection of free sgCas9-AdV or sgCas9-AdV/Gel. Data is presented as mean ± SD. (n = 3). Statistical significance was calculated by two-tailed unpaired Student's t test, $^{**}P < 0.01$.

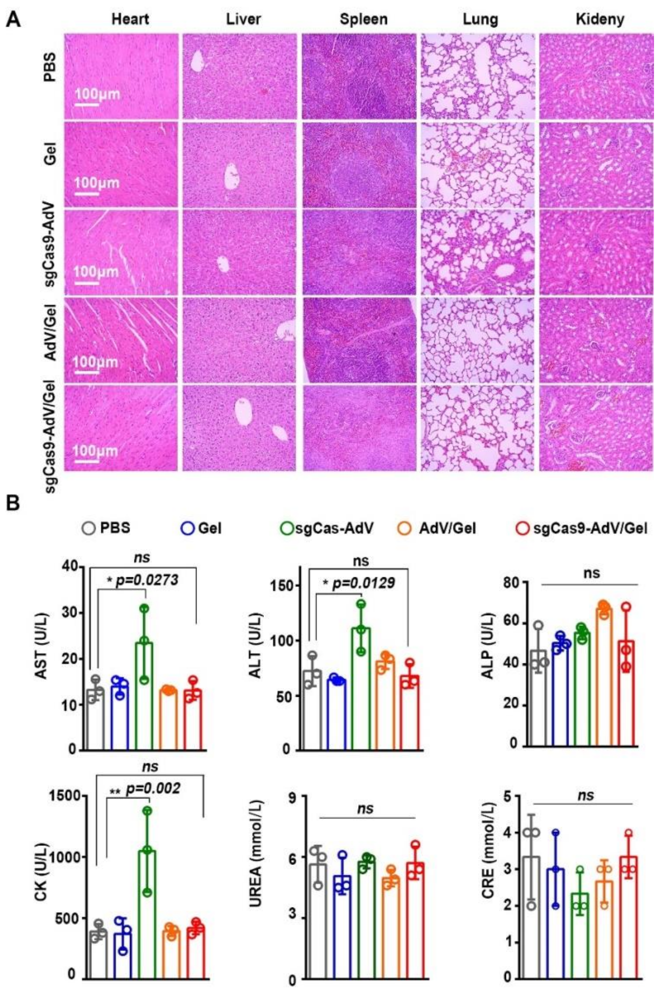

Figure S11. (A) Histological H&E staining of major organs after indicated treatments. Scale bar: 100 μm. (B) Blood biochemistry after indicated treatments. Data is presented as mean ± SD. (n = 3). Statistical significance was calculated by one-way ANOVA test, **P* < 0.05, ***P* < 0.01 and ****P* < 0.001. ns means no significant difference.

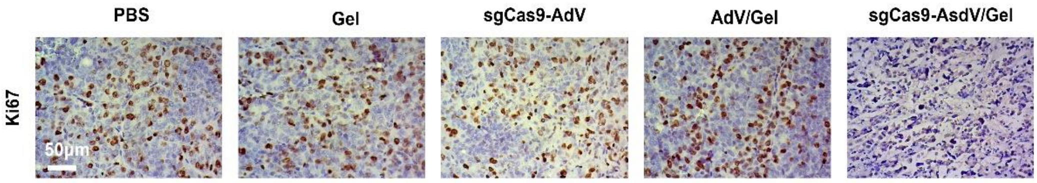

Figure S12. Representative images of tumor slices stained with Ki67 after indicated treatments.

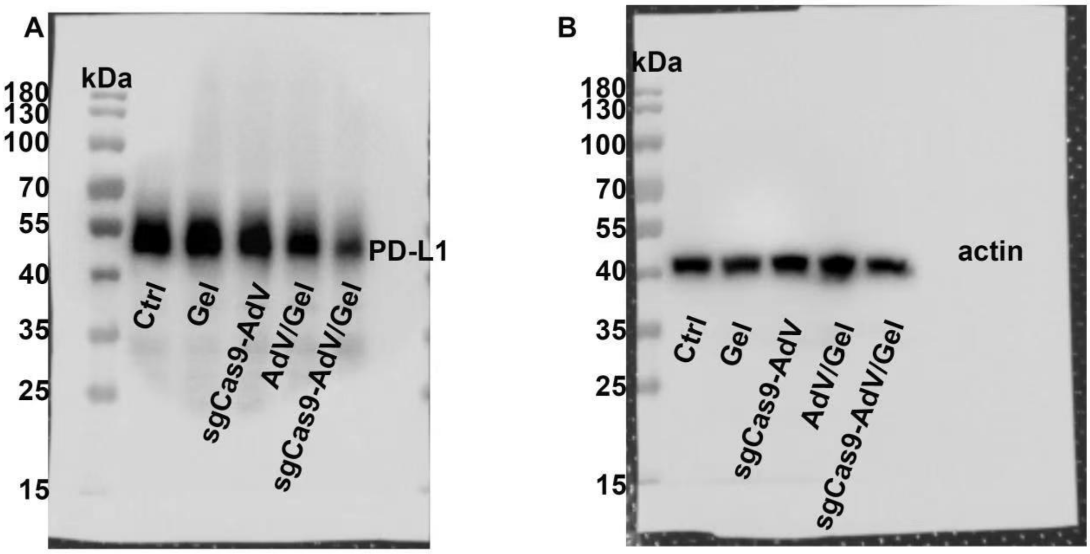

Figure S13. Full gel scan image of Figure 3I.

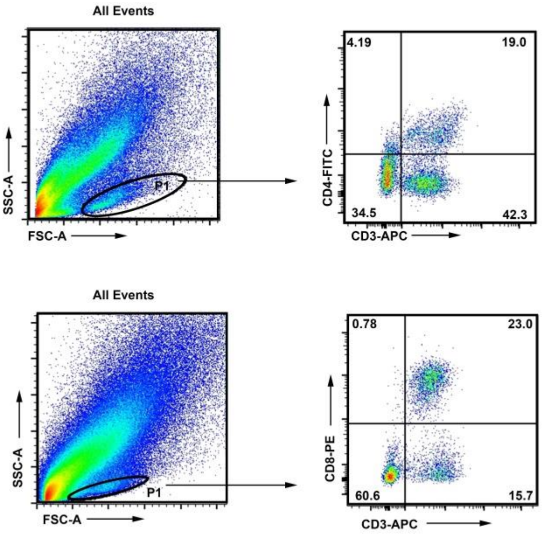

Figure S14. Representative flow cytometry gating strategies for experiments in Figure 4A and B. To detect the percentage of $CD3^+CD4^+$ and $CD3^+CD8^+$ T cells in the Hepa1-6 tumor.

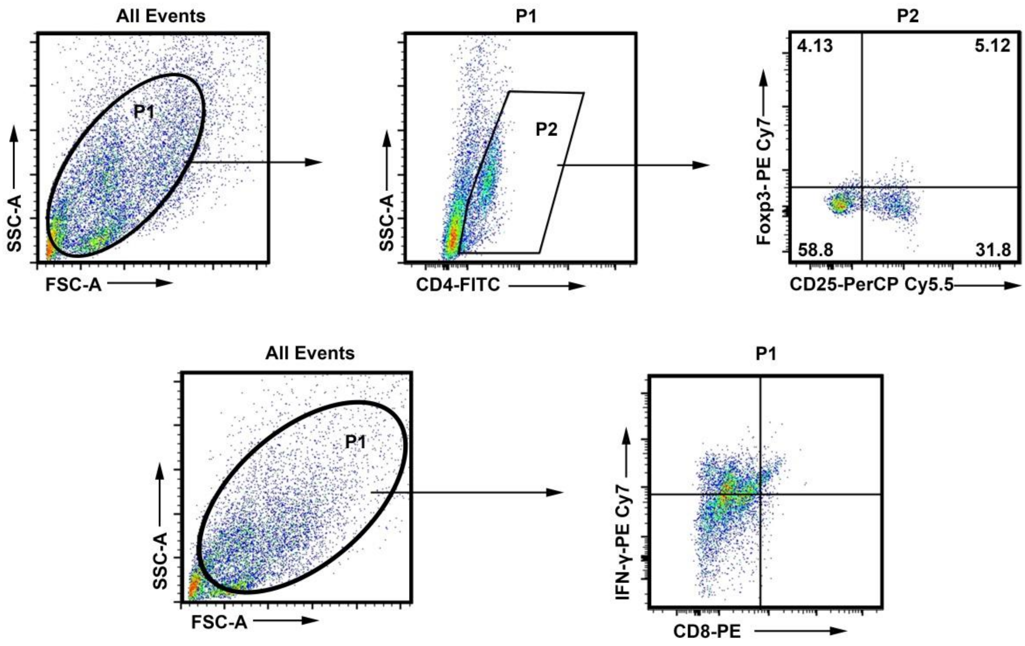

Figure S15. Representative flow cytometry gating strategies for experiments in Figure 4C and D. To detect the percentage of Treg and IFN-γ^{+}CD8^{+} T cells in the Hepa1-6 tumor.

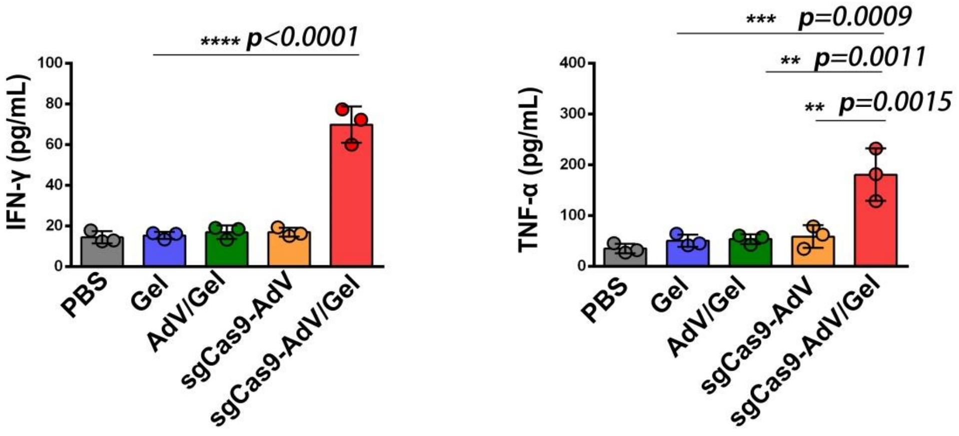

Figure S16. Cytokine levels of INF-γ and TNF-α in tumor lysates by ELISA analysis after indicated treatments (n=3). Statistical significance was calculated by one-way ANOVA with Tukey's post hoc test.

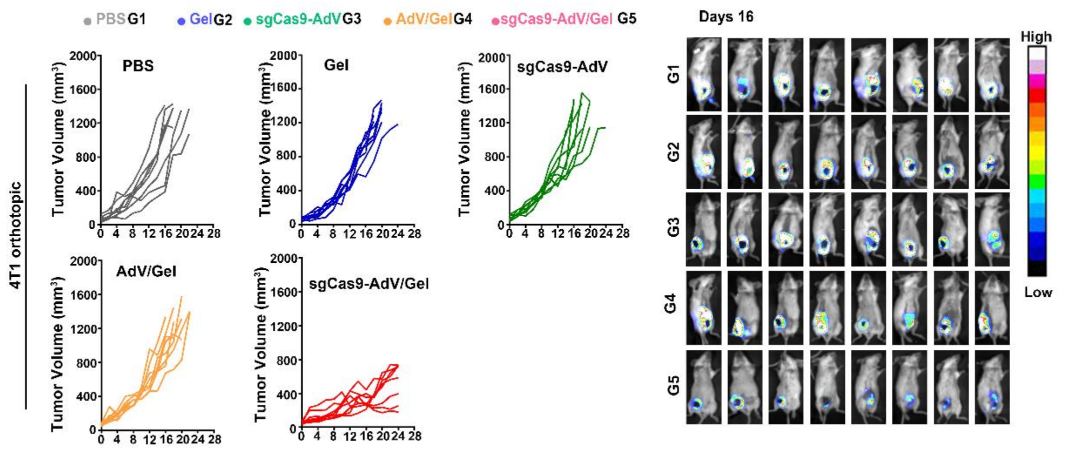

Figure S17. Individual tumor growth kinetics and bioluminescence imaging of Luc-4T1 tumor bearing mice on the day 16 after different treatments.

Reference

1 X. Zhang, C. Xu, S. Gao, P. Li, Y. Kong, T. Li, Y. Li, F. -J. Xu, J. Du, *Adv. Sci*. **2019**, *6*, 1900386.

2 Q. Zhao, Y. Wang, B. Zhao, H. Chen, Z. Cai, Y. Zheng, Y. Zeng, D. Zhang, X. Liu, *Nano Lett*. **2022**, *22*, 2048.

3. A. Dobin, C. A. Davis, F. Schlesinger, J. Drenkow, C.Zaleski, S. Jha, P. Batut, M. Chaisson, T. R. Gingeras, *Bioinformatics* **2013**, *29*, 15.

4 C. Trapnell, A. Roberts, L. Goff, G. Pertea, D. Kim, D. R. Kelley, H. Pimentel, S. L. Salzberg, J. L. Rinn, L. Pachter, *Nat. Protoc*. **2012**, *7*, 562.
